# Synthesis and Characterization of a Novel Sol–Gel-Derived Ni-Doped TiO_2_ Photocatalyst for Rapid Visible Light-Driven Mineralization of Paracetamol

**DOI:** 10.3390/nano15070530

**Published:** 2025-03-31

**Authors:** Nicola Morante, Katia Monzillo, Vincenzo Vaiano, Zukhra C. Kadirova, Diana Sannino

**Affiliations:** 1 Department of Industrial Engineering, University of Salerno, Via Giovanni Paolo II 132, 84084 Fisciano, SA, Italy; nmorante@unisa.it (N.M.); kmonzillo@unisa.it (K.M.); vvaiano@unisa.it (V.V.); 2 Uzbekistan–Japan Innovation Center of Youth, University Street 2B, Tashkent 100095, Uzbekistan; zuhra_kadirova@yahoo.com

**Keywords:** heterogeneous photocatalysis, paracetamol, visible light driven, slurry photoreactor, sol-gel synthesis, catalyst stability, pharmaceutical pollutants, Ni-TiO_2_

## Abstract

The increasing presence of pharmaceutical contaminants, such as paracetamol, in water sources necessitates the development of efficient and sustainable treatment technologies. This study investigates the photocatalytic degradation and mineralization of paracetamol under visible light using nickel-doped titanium dioxide (Ni–TiO_2_) catalysts synthesized via the sol-gel method. The catalysts were characterized through Raman spectroscopy, UV–Vis diffuse reflectance spectroscopy (UV–Vis DRS), and surface area measurements. Ni doping enhanced the visible light absorption of TiO_2_, reducing its band gap from 3.11 eV (undoped) to 2.49 eV at 0.20 wt.% Ni loading, while Raman analysis confirmed Ni incorporation with anatase as the predominant phase. The Ni(0.1%)-TiO_2_ catalyst exhibited the highest photocatalytic activity, achieving 88% total organic carbon (TOC) removal of paracetamol (5 ppm) after 180 min under optimal conditions (catalyst dosage, 3 g L^−1^). Stability tests demonstrated 84% retained efficiency over five cycles, with a kinetic rate constant of 0.010 min^−1^. Hydroxyl radicals were identified as the main reactive species. The catalyst maintained high performance in tap water, achieving 78.8% TOC removal. These findings highlight the potential of Ni(0.1%)-TiO_2_ as a cost-effective, visible light-active photocatalyst for the removal of pharmaceutical pollutants, with promising scalability for industrial water treatment applications.

## 1. Introduction

The contamination of aquatic environments with pharmaceutical compounds is a growing environmental concern. Among these, paracetamol (acetaminophen) is one of the most widely consumed analgesics and antipyretics, with global usage continuing to rise due to its over-the-counter availability and broad therapeutic applications [1,2].

Paracetamol often enters water systems primarily through human excretion, industrial effluents, and improper disposal [3]. Conventional wastewater treatment plants are typically unable to fully degrade or remove pharmaceutical compounds, resulting in their persistent presence in surface water, groundwater, and, in some cases, drinking water supplies [4,5].

This persistence raises serious ecological and public health concerns, as exposure to paracetamol can disrupt aquatic ecosystems and potentially lead to bioaccumulation, posing risks to human health [6,7].

Advanced oxidation processes (AOPs), such as photocatalysis, are considered promising technologies for the removal of recalcitrant pollutants like paracetamol [8,9,10].

AOPs rely on the generation of highly reactive species, such as hydroxyl radicals (OH^•^), which can oxidize organic pollutants into harmless end products like water and carbon dioxide [11,12]. Among AOPs, photocatalysis has gained particular attention due to its potential for low-cost and sustainable application in water treatment systems [13,14,15]. Photocatalysis typically utilizes semiconductor materials to harness light energy for the degradation of contaminants. Titanium dioxide (TiO_2_) is the most extensively studied photocatalyst owing to its strong oxidative power, high stability, and non-toxic nature [16,17,18].

Despite its advantages, TiO_2_ faces inherent limitations that hinder its broad application. With a bandgap of approximately 3.2 eV, TiO_2_ can only be activated by ultraviolet (UV) light, which accounts for a mere 4–5% of sunlight [19,20]. This restricts its efficiency under natural solar irradiation. Furthermore, TiO_2_ photocatalytic systems suffer from the rapid recombination of photogenerated electron-hole pairs, which reduces the availability of reactive species for pollutant degradation [21,22].

To overcome these challenges, significant research efforts have focused on modifying TiO_2_ through doping with metal and non-metal elements [23,24]. Research on synthesized metal-doped titanium dioxide (TiO_2_) materials is extensive, especially for improving photocatalytic, electrical, and optical characteristics. By altering its band structure, charge carrier dynamics, and surface characteristics, transition metal doping into TiO_2_ makes it appropriate for uses such as environmental remediation photocatalysis [25,26,27]. The incorporation of transition metals such as nickel (Ni) into the TiO_2_ lattice has shown a potential to enhance photocatalytic performance. Nickel doping introduces impurity levels within the TiO_2_ bandgap, enabling the absorption of visible light, which constitutes a much larger fraction of the solar spectrum [28,29]. Additionally, nickel acts as a charge-trapping site, reducing the recombination of electron-hole pairs and increasing the lifetime of reactive species [30,31]. These modifications can significantly enhance the efficiency of TiO_2_-based systems under natural sunlight. Common methodologies for synthesizing Ni-doped TiO_2_, with a focus on transition metal incorporation techniques, are sol-gel, hydrothermal, solvothermal, co-precipitation, impregnation, flame spray pyrolysis (FSP), and solid-state reaction methods [32]. Because of its ease of use, low synthesis temperature, and molecular-level composition control, the sol-gel procedure is one of the most popular ways to dope TiO_2_ with transition metals [16,33]. In order to create the final crystalline doped TiO_2_ phase, the process first hydrolyses and condenses titanium precursors while adding metal dopants in the form of metal salts or organometallic compounds. This is typically followed by gelation, drying, and calcination [34]. At low processing temperatures, it enables control over particle size and shape as well as uniform dopant dispersion. Nevertheless, at high doping concentrations, it may cause metal segregation or precipitation, and post-synthesis heat treatment is required to improve crystallinity [35]. During hydrothermal synthesis, precursors are reacted in a temperature-controlled, high-pressure aqueous solution, and this process is extended to non-aqueous solvents during solvothermal synthesis [36]. In an acidic or basic solution, titanium and metal dopant precursors (such as metal chlorides or nitrates) are combined. For regulated crystal development, the solution is heated to temperatures between 100 and 250 °C in a sealed autoclave. The resulting nanoparticles are gathered, cleaned, and allowed to dry. Because of the high-pressure settings, it produces good crystallinity and phase purity, as well as regulated particle size and morphology. It is also distinguished by the increased solubility of metal dopants. However, it is time-consuming and energy-intensive and requires careful tuning of reaction conditions [37]. The co-precipitation approach uses alkaline agents, such as NH_4_OH or NaOH, to promote the precipitation of hydroxides while simultaneously precipitating metal dopants and TiO_2_ from a precursor solution. To obtain doped TiO_2_, the precipitate is filtered, cleaned, dried, and calcined. It is easy to use, economical, and appropriate for large-scale manufacturing. Poor control over dopant distribution and the potential for secondary phase formation, if not optimized, are the primary disadvantages [38]. A metal dopant is applied to pre-formed TiO_2_ surfaces during impregnation, and the materials are then calcined. It is ideal for maintaining the TiO_2_ crystal structure in surface doping, but catalytic applications are less efficient when doping in mass is required. In addition, agglomeration of metal ions on the surface is possible [39]. FSP is a high-temperature synthesis technique that uses a flame to atomize a precursor solution to create nanoparticles. It is a high-purity, quick synthesis that can be scaled up for industry. Controlling dopant concentration and dispersion is challenging, though, and calls for specific tools [32]. TiO_2_ and metal dopant precursors are instead mechanically mixed and heated in a high-temperature process known as a solid-state reaction. It can result in inhomogeneous doping and phase segregation, and the high-temperature processing may cause grain growth, despite being straightforward and scalable for large-scale manufacturing [40]. The choice of method depends on the desired application, dopant concentration, and structural properties needed. For low dopant concentrations, unique aspects and potential advantages of the sol-gel method include [41]: (i) superior dopant homogeneity, due to the molecular-level dispersion of transition metal dopants, reducing phase segregation; (ii) control over morphology and low particle size, since fine-tuning synthesis conditions enables precise control of nanostructure formation; (iii) low-temperature processing—unlike solid-state reactions or flame spray pyrolysis, sol-gel operates at relatively low temperatures, reducing energy consumption; (iv) high purity and phase control, obtained by precursor selection and calcination conditions to minimize secondary phase formation; (v) versatility for tailored properties, to introduce additional functionalities (e.g., mesoporosity, core-shell structures). However, it requires additional calcination to remove residual organics and could have scalability issues, which can be overcome by adapting continuous sol-gel or hybrid methods for large-scale production [42]. Thus, the sol-gel method stands out as a highly versatile and precise synthesis approach for metal-doped TiO_2_, particularly when uniform doping, controlled morphology, and phase purity are critical.

Ni-doped TiO_2_ (Ni-TiO_2_) has demonstrated effectiveness in degrading various organic pollutants, including dyes, pesticides, and phenolic compounds [43,44,45,46]. Paracetamol degradation presents unique challenges due to its high solubility, chemical stability, and the potential formation of toxic byproducts during incomplete oxidation [47]. In recent years, studies have highlighted the significance of optimizing the doping concentration and operational parameters to achieve maximum photocatalytic efficiency [48,49,50]. Factors such as catalyst loading, light intensity, pH, and the initial pollutant concentration play critical roles in determining the degradation efficiency. Additionally, understanding the reaction mechanisms and the formation of intermediate byproducts is essential to ensure the safe and complete mineralization of pharmaceutical contaminants.

This study aims to evaluate the photocatalytic performance of Ni-TiO_2_ photocatalyst for the mineralization of paracetamol in aqueous solutions. The research investigates the effects of nickel doping on the structural, optical, and photocatalytic properties of TiO_2_. Furthermore, the work focuses on optimizing key reaction parameters to enhance the degradation efficiency under visible light irradiation. The kinetics and mechanisms of paracetamol mineralization are analyzed to provide insights into the potential practical applications of Ni-TiO_2_ in advanced water treatment systems. By addressing current knowledge gaps, this study contributes to the development of more efficient and sustainable photocatalytic technologies for mitigating pharmaceutical pollution in aquatic environments.

## 2. Materials and Methods

### 2.1. Materials

Titanium tetraisopropoxide (C_12_H_28_O_4_Ti ≥ 97% (*w*/*w*), Sigma Aldrich Italia s.r.l., Milano, Italy), nickel(II) acetate tetrahydrate (Ni(CO_2_CH_3_)_2_∙4 H_2_O ≥ 98% (*w*/*w*), Sigma Aldrich), acetaminophen (CH_3_CONHC_6_H_4_OH, Sigma Aldrich), hydrogen peroxide solution (H_2_O_2_, 30% (*w*/*w*) in H_2_O, Sigma Aldrich), titanium(IV) oxysulfate solution (TiOSO_4_ ~15% (*w*/*w*) in dilute sulfuric acid ≥ 99.99%, Sigma Aldrich), sodium hydroxide (NaOH, pellets anhydrous, ≥ 98% (*w*/*w*), Sigma Aldrich), hydrochloric acid solution (HCl, 37% (*w*/*w*) in H_2_O, Carlo Erba, Milano, Italy), and MilliQ water (Millipore) were purchased and used as received.

### 2.2. Preparation of Photocatalytic Nanoparticles

TiO_2_ and Ni-doped TiO_2_ photocatalysts were synthesized using a wet chemical method, following the procedure described in prior research [51,52,53]. Nickel(II) acetate tetrahydrate was used as the nickel source, while titanium tetraisopropoxide (TTIP) served as the titanium precursor. This synthesis strategy facilitated the incorporation of nickel ions into the TiO_2_ lattice, enhancing the photocatalytic properties by introducing metal dopant ions. TTIP was added dropwise into an aqueous solution containing a fixed concentration of nickel(II) acetate tetrahydrate under constant stirring at room temperature for 10 min to ensure thorough mixing and homogeneity. The resulting suspensions were centrifuged to isolate the precipitates, which were washed three times with distilled water to remove impurities. The cleaned precipitates were then calcined in a muffle furnace at 450 °C for 30 min in static air. The final photocatalysts were labeled as Ni(x%)-TiO_2_, where x denotes the nominal weight percentage of nickel incorporated. Table 1 provides details on the volumes of solutions, the quantities of Ti and Ni precursors used, the Ni/Ti molar ratios, and the nominal nickel content of the synthesized samples.

### 2.3. Characterization of the Photocatalysts

The Brunauer–Emmett–Teller (BET) surface area of the catalysts was measured through dynamic nitrogen adsorption at –196 °C using a Costech Sorptometer 1042. Before the analysis, the samples were pretreated in a helium flow at 150 °C for 30 min to remove any adsorbed impurities.

Raman spectroscopy was conducted at room temperature using a Dispersive MicroRaman spectrometer (InVia, Renishaw Italia, Pianezza Torino, Italy) equipped with a 514 nm excitation laser. The spectra were collected over the Raman shift range of 100–1200 cm^−1^ to identify the structural and vibrational characteristics of the materials.

SEM and EDX analysis of Ni0.1-TiO_2_ was performed using a Phenom ProX microscope operating in high-vacuum mode, coupled with an EDX probe for elemental analysis, following preliminary gold sputtering of the sample.

UV–Vis diffuse reflectance spectroscopy (UV–Vis DRS) was performed with a PerkinElmer Lambda 35 spectrophotometer fitted with an RSA-PE-20 reflectance accessory (Labsphere Inc., North Sutton, NH, USA). The band gap energies of the nanocomposites were determined by applying the Kubelka–Munk (K-M) function. Specifically, plots of (K-M × h ν)^0.5^ against h ν were used to estimate the optical band gaps [54].

The point of zero charge (PZC) of the samples was determined using the mass titration method, as outlined by Noh and Schwarz (1989) [55]. To reduce the risk of sample dissolution, shorter stabilization times of 2 h were employed after each powder addition. The PZC was defined as the pH at which the net surface charge of the material equaled zero.

### 2.4. Experimental Setup for Photocatalytic Activity Tests

**Figure 1:**
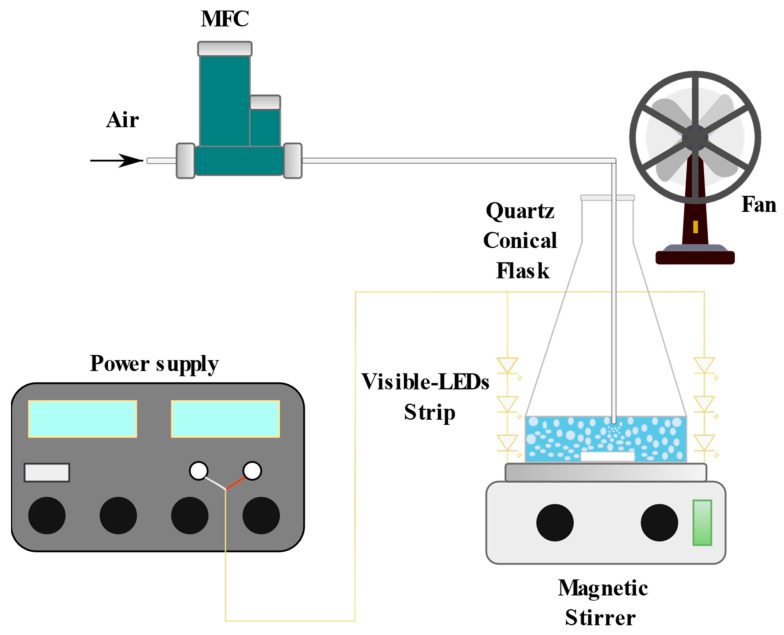
Picture of the experimental apparatus scheme used for the paracetamol degradation tests.

Photocatalytic activity tests were conducted to evaluate the mineralization of paracetamol in a batch photoreactor system. The experimental setup consisted of a quartz conical flask with an internal diameter of 8 cm and a height of 15 cm. The test solution (50 mL) was prepared by dissolving the required quantity of paracetamol in distilled water. Experiments were performed at room temperature with initial contaminant concentrations ranging from 5 to 15 ppm, photocatalyst dosages between 1.5 and 4.5 g L^−1^, and initial pH values spanning 3 to 9. Magnetic stirring was maintained at 300 rpm and an air distributor was set to a flow rate of 142 Ncc min^−1^ throughout the experiments to ensure homogeneous mixing. Irradiation was provided by a visible-LED strip (nominal power: 10 W, supplied by LED Lighting Hut) with an emission range of 400–700 nm and a light intensity of 13 mW cm^−2^. The LED strip was positioned in direct contact with the external surface of the photoreactor, ensuring uniform irradiation of the solution volume. To mitigate thermal effects induced by the lamps, a fan was placed adjacent to the photoreactor for cooling, and aluminum foil was used to shield the setup from light dispersion. A schematic diagram of the apparatus is presented in Figure 1.

The experimental procedure consisted of an initial 2-h dark equilibration phase to establish adsorption–desorption equilibrium between the pharmaceutical molecules and the photocatalyst surface. During this phase, the temperature was monitored and recorded using a Greisinger G 1720 digital thermometer, registering approximately 25 °C when the LEDs were off. Following this, the irradiation phase began by activating the photocatalyst through lamp illumination. The temperature increased to around 35 °C when the LEDs were activated. Samples (3 mL) were collected at predetermined intervals during both the dark and illuminated phases to monitor the adsorption and subsequent degradation of paracetamol. The contaminant concentration was quantified spectrophotometrically using a UV–Vis spectrophotometer (Thermo Scientific Evolution 201, Waltham, MA, USA) at the wavelength corresponding to paracetamol’s maximum absorbance (245 nm) [56].

Mineralization of the pollutant and its degradation intermediates was evaluated by measuring the total organic carbon (TOC) content of the samples. TOC analysis involved CO_2_ generation through high-temperature catalytic combustion using a Pt/Al_2_O_3_ catalyst in a fixed-bed reactor operating at 680 °C [57]. The kinetics of paracetamol degradation were modeled using the Langmuir–Hinshelwood kinetic framework (Equation (1)).

(1) $$r=\frac{k_{r}K_{ad}c}{1+K_{ad}c}$$

where *k*_r_, *K*_ad_, and *c* are the kinetic degradation constant, adsorption equilibrium constant, and paracetamol concentration, respectively.

Under conditions of experimental test, with low concentration of the contaminant, the expression simplifies to a first-order kinetic equation:

(2) $$r=k_{r}K_{ad}c=kc$$

To determine the apparent kinetic constant (*k*) for paracetamol degradation, a mass balance was applied to the reaction system. The calculation was based on the following integrated rate equation:

(3) $$F\left(t\right)=-\ln\left(\frac{c}{c_{0}}\right)=kt$$

Analogously, assuming first-order kinetics for mineralization, the apparent mineralization constant (*k*_min_) was derived from the following equation:

(4) $$G\left(t\right)=-\ln\left(\frac{TOC}{{TOC}_{0}}\right)=k_{min}\;t$$

The slopes of plots of −ln(*c/c*_0_) and −ln(TOC/TOC_0_) versus time provided values for *k* and *k*_min_, respectively. TOC removal (mineralization) and paracetamol degradation efficiency were calculated using the following relationships:

(5) $$TOC\;Removal\;Efficiency\left(t\right)=\left(1-\frac{TOC}{TOC_{0}}\right)\times 100$$

(6) $$Degradation\;Efficiency\left(t\right)=\left(1-\frac{c}{c_{0}}\right)\times 100$$

where TOC(t) and *c*(*t*) represent TOC and paracetamol concentrations at a given irradiation time, and TOC_0_ and *c*_0_ are the corresponding initial values.

### 2.5. Evaluation of Possible Nickel Leaching

The extent of nickel leaching from the Ni-doped TiO_2_ photocatalysts was evaluated using a simple spectrophotometric method based on the formation of a Ni(II)-EDTA complex [58,59,60,61]. In this method, any nickel ions (Ni^2+^) potentially released into the solution form a complex with ethylenediaminetetraacetic acid (EDTA). The resulting [Ni(II)-EDTA]^2−^ complex exhibits a characteristic absorbance at 394 nm [61], allowing for rapid and sensitive detection without the need for direct nickel concentration measurements. This approach provided valuable insights into catalyst stability under photocatalytic conditions and the potential release of nickel into the solution during the degradation process.

After three hours of visible light irradiation, the reaction suspension was centrifuged at 5000 rpm for 3 min to separate the photocatalyst from the solution. A fixed amount of EDTA was then added to 40 mL of the solution to enable the potential formation of the [Ni(II)-EDTA]^2−^ complex, according to the following reaction:

(7) $$Ni^{2+}+EDTA\to {\left[Ni-EDTA\right]}^{2^{-}}+2H^{+}$$

The mass of EDTA added was calculated to be twice the theoretical molar amount of nickel that could be released by the samples to ensure complete complexation. Subsequently, 2 mL of the solution was analyzed spectrophotometrically to detect any possible complex formation, thereby confirming or excluding nickel leaching.

### 2.6. Band Structure Estimation

The photocatalytic performance of semiconductor materials is intrinsically governed by their electronic band structure, particularly the positions of the conduction band (CB) and valence band (VB), which dictate the redox potential of photogenerated charge carriers. These energetic parameters play a critical role in driving oxidation and reduction processes during photocatalytic degradation of organic contaminants. The relative positions of the CB and VB edges can be theoretically estimated using the Mulliken electronegativity approach, which offers a practical method to predict the redox capability of a photocatalyst. According to this method, the valence band edge potential (*E*_VB_) and conduction band edge potential (*E*_CB_) are calculated using the following expressions [62,63]:

(8) $$E_{VB}=\chi -E_{c}+\frac{1}{2}E_{bg}-0.059\times 7$$

(9) $$E_{CB}=E_{VB}-E_{bg}$$

where χ represents the absolute electronegativity of the semiconductor, typically defined as the geometric mean of the electronegativities of its constituent elements; *E*_c_ denotes the energy of free electrons on the hydrogen scale (approximately 4.5 eV); and *E*_bg_ is the energy band gap of the photocatalyst, usually determined experimentally via UV–Vis diffuse reflectance spectroscopy. The pH-dependent term accounts for the influence of solution acidity on the VB potential. This theoretical estimation provides a valuable framework for correlating the electronic structure of photocatalysts with their photocatalytic activity and evaluating their suitability for specific redox-driven reactions.

## 3. Results and Discussion

### 3.1. Photocatalysts Characterizations

The specific surface area of the synthesized photocatalysts was determined using nitrogen adsorption–desorption isotherms via BET analysis, as photocatalysis is inherently an adsorption-driven process [64]. In this context, pollutant molecules act as adsorbates, while the photocatalyst surface functions as the adsorbent, making a sufficiently large surface area essential for efficient photocatalytic activity [64,65]. Evaluating the surface area of the developed photocatalyst materials is therefore crucial, as it provides a basis for comparing the inherent and compositional surface characteristics of the samples. The incorporation of Ni within the TiO_2_ crystal lattice was observed to enhance the specific surface area of the doped photocatalysts. The measured surface area values are summarized in Table 2.

The undoped TiO_2_ exhibited an intrinsic surface area of 101 m^2^ g^−1^. Upon Ni incorporation, the surface area showed a slight but progressive increase with rising nickel content, ranging from 102 to 104.5 m^2^ g^−1^. This enhancement in surface area suggests a positive influence of Ni doping on surface characteristics, which likely contributes to the improved adsorption efficiency of samples.

**Figure 2:**
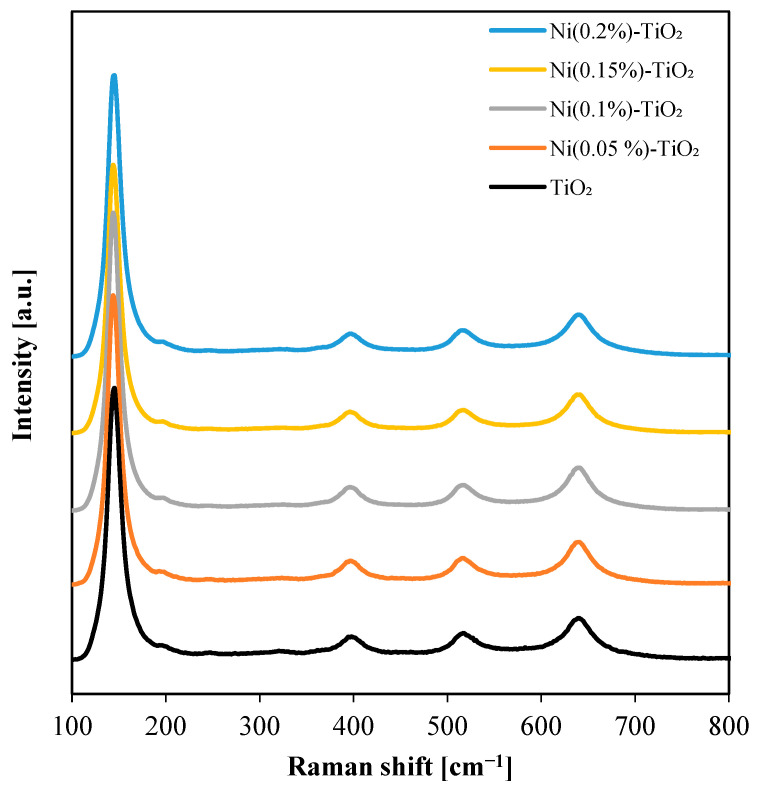
Raman spectra of the undoped and Ni-doped TiO_2_ photocatalysts.

The structural properties of undoped and Ni-doped TiO_2_ were further analyzed using Raman spectroscopy. Spectra recorded in the range of 100–800 cm^−1^ are presented in Figure 2. Characteristic bands were observed at 638, 516, 397, 195, and 144 cm^−1^, indicating that the predominant polymorph was anatase, with traces of brookite [52,66]. The main anatase-phase bands appeared at 145.3 cm^−1^ and 639.7 cm^−1^, corresponding to the Eg(1) and Eg(3) vibrational modes, respectively, associated with symmetric stretching vibrations of the O–Ti–O bonds [67]. The band at 396.5 cm^−1^ was assigned to the B1g(1) mode, which is attributed to symmetric bending vibrations of the O–Ti–O bonds [44,67]. Additionally, the peak at 517.8 cm^−1^ was associated with the A1g + B1g modes, where the A1g mode corresponds to antisymmetric bending vibrations of O–Ti–O bonds [44,67]. A weak signal at 707 cm^−1^, close to the detection limit, may suggest the presence of trace amounts of NiTiO_3_, although its contribution was minimal [54].

**Figure 3:**
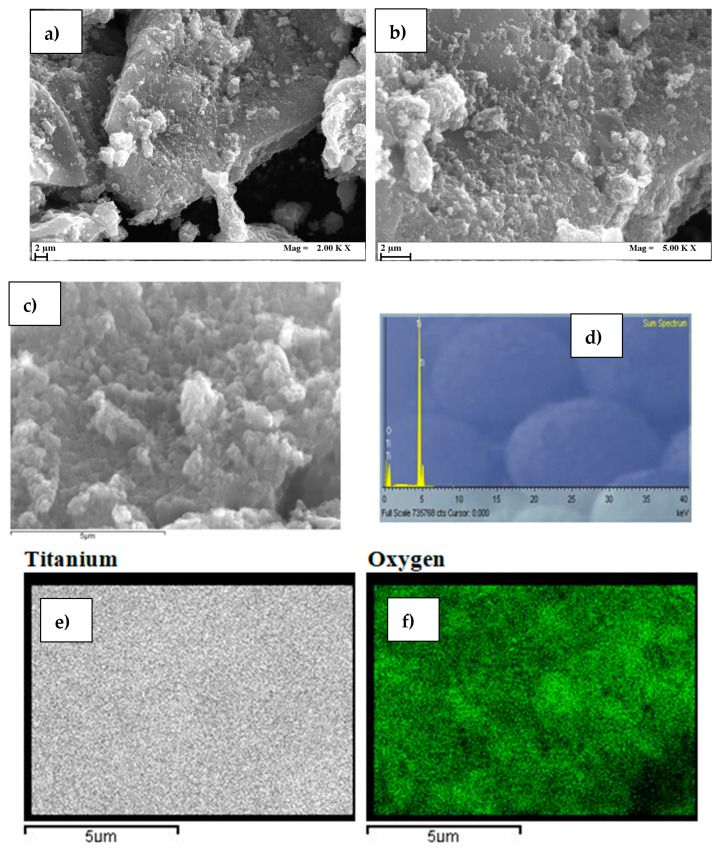
SEM and EDX of Ni(0.1%)-TiO_2_ photocatalyst: (**a**–**c**) three different magnifications of the aggregates, (**d**) spectrum sum, (**e**) titanium distribution, (**f**) oxygen distribution.

Grainy aggregates of Ni(0.1)-TiO_2_ can be observed in Figure 3a–c at different magnifications by SEM morphological analysis. Rounded particles are dispersed on larger pieces of irregular geometry. However, EDX analysis (Figure 3d,f) revealed only Ti and oxygen, even after prolonged acquisition, despite their uniform distribution across the aggregated particle surface. This could indicate that Ni is either present in a very low amount or is well embedded within the crystal structure of TiO_2_. The latter hypothesis would confirm the successful incorporatoin of Ni into the titanium dioxide structure.

**Figure 4:**
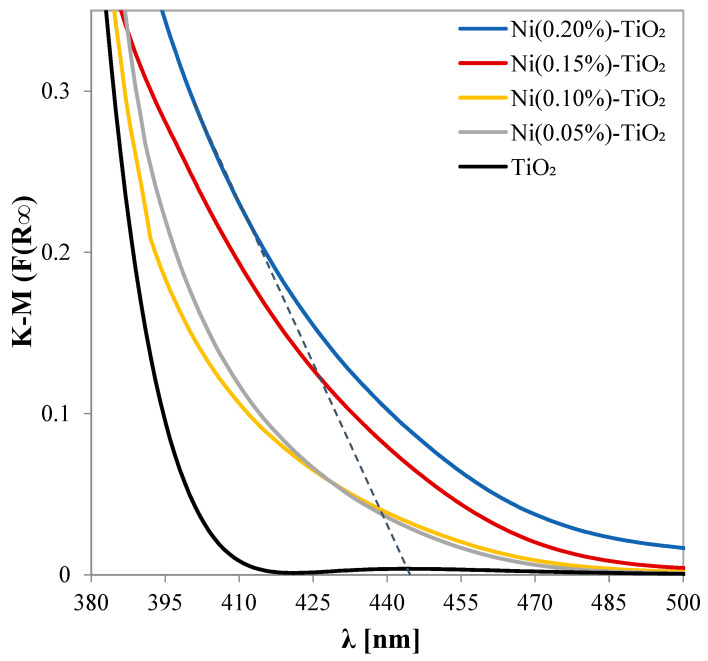
Kubelka–Munk spectra of TiO_2_ and Ni-doped TiO_2_ photocatalysts.

The UV–Vis absorbance spectra of Ni-doped TiO_2_ nanopowders in the range of 380–500 nm are shown in Figure 4.

It is clear that the Ni-doped TiO_2_ samples exhibit an absorption onset shifted from 400 (for pure TiO_2_) to 445 nm, suggesting that Ni-doped TiO_2_ nanoparticles can be used for photocatalytic degradation of organic compounds under visible light irradiation. This is a consequence of the doping effect that narrows the band gap by forming a mini-band below the conduction band, extending the absorption wavelength range into the visible light range [68,69]. The indirect energy band gap (*E*_bg_) of the as-prepared samples was determined, starting from the indirect transition of the TiO_2_ semiconductor (Figure S1). It reveals a band gap of the Ni-doped TiO_2_ nanoparticles lower than that of the TiO_2_-anatase phase [29,68], recording *E*_bg_ values of 3.11 eV for TiO_2_, and 2.53, 2.50, 2.44, and 2.49 for 0.05, 0.10, 0.15, and 0.2% of Ni-doped TiO_2_ (Table 2). This narrower band gap made the electron more easily excitable from the valence band to the conduction band, enhancing the photocatalytic activity of the doped samples.

The surface acidity of the synthesized samples was evaluated using the mass titration method. The pH values corresponding to the point of zero charge (PZC) are presented in Table 2. The undoped TiO_2_ exhibited a PZC value of 6.02, which is consistent with literature values for TiO_2_ in the anatase phase [70]. In contrast, Ni-doped photocatalysts demonstrated an increasingly acidic character, with PZC values of 5.75, 5.47, 5.18, and 4.95 for nickel loadings of 0.05%, 0.1%, 0.15%, and 0.2% by weight, respectively. This trend highlights the influence of Ni incorporation on the surface properties of the photocatalyst.

### 3.2. Photocatalytic Activity Tests

#### 3.2.1. Screening Tests

**Figure 5:**
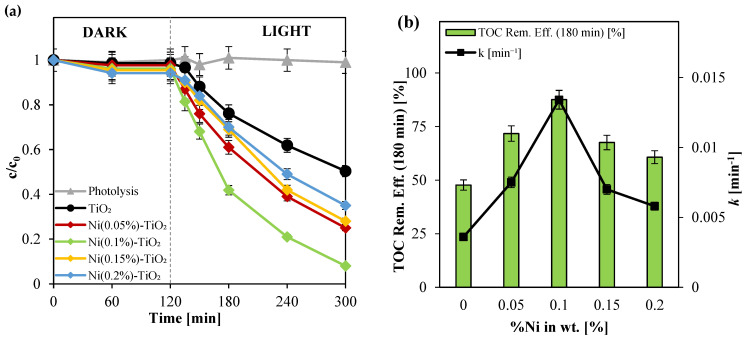
(**a**) Photocatalytic paracetamol degradation for direct photolysis and Ni-doped TiO_2_ photocatalysts; (**b**) TOC removal efficiency and apparent paracetamol discoloration kinetic constants for Ni-Doped TiO_2_ photocatalysts after 180 min of visible light irradiation.

Initially, a direct photolysis test of paracetamol was conducted. As shown in Figure 5, the concentration of paracetamol remained approximately unchanged after 120 min of irradiation. This negligible degradation is likely attributed to the weak absorption of paracetamol within the spectral range of 300 to 800 nm [71]. Subsequently, screening tests were performed to determine the optimal nickel doping level for TiO_2_-based photocatalysts in the degradation and mineralization of paracetamol at an initial concentration of 5 ppm. The evaluated photocatalysts included undoped TiO_2_, Ni(0.05%)-TiO_2_, Ni(0.10%)-TiO_2_, Ni(0.15%)-TiO_2_, and Ni(0.20%)-TiO_2_, with each test conducted at a photocatalyst dosage of 3 g L^−1^. In particular, for the nickel-doped titania samples, nickel leaching into the solution was assessed using the colorimetric method described in Section 2.5. No detectable leaching was observed in any sample, indicating effective incorporation of nickel within the titania crystal lattice, as corroborated by structural and compositional characterizations.

Among the tested photocatalysts, Ni(0.10%)-TiO_2_ exhibited the highest photocatalytic activity (Figure 5). Specifically, after 180 min of visible light irradiation, the degradation efficiency of paracetamol and total organic carbon (TOC) removal efficiency reached approximately 92% and 88%, respectively. In contrast, the other Ni-doped photocatalysts: Ni(0.05%)-TiO_2_, Ni(0.15%)-TiO_2_, and Ni(0.20%)-TiO_2_, achieved degradation efficiencies of approximately 72%, 68%, and 61%, respectively, under the same conditions. Undoped TiO_2_ demonstrated the lowest photocatalytic performance, achieving only 49% paracetamol degradation after 180 min of irradiation. These results indicate that nickel doping enhances the photocatalytic activity of TiO_2_, allowing the activation of titania with visible light and limiting the recombination of the generated photoexcited pairs [72,73]. However, as mentioned in the literature, excessive dopant load could act as recombination centers of photo-induced electrons and holes, thereby decreasing photocatalytic activity [74,75,76]. Therefore, Ni(0.10%)-TiO_2_ was identified as the optimal photocatalyst, and subsequent experiments were conducted using this doping load.

#### 3.2.2. Stability Tests

**Figure 6:**
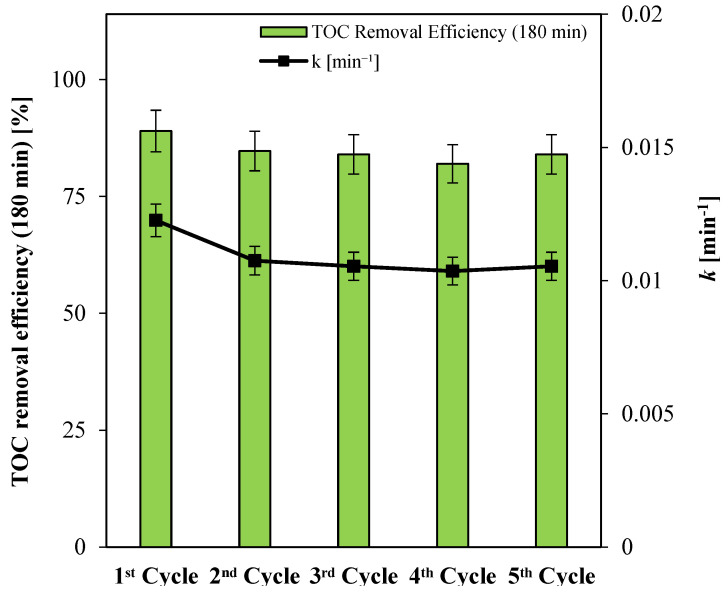
Paracetamol TOC removal efficiency after 180 min under visible light, and apparent kinetic constant of degradation (*k*) values obtained by the stability tests.

Fifth-cycle stability tests were conducted with the Ni(0.1%)-TiO_2_ photocatalyst under the same operating conditions as the initial screening experiments (Figure 6). The results revealed a slight initial deactivation of the photocatalyst, evident in both the degradation and mineralization efficiencies for paracetamol. However, by the second cycle, the photocatalytic activity stabilized at a slightly reduced level. Notably, the mineralization efficiency was maintained at approximately 84%, with an apparent kinetic constant for paracetamol degradation of 0.010 min^−1^ after 180 min of visible light irradiation. These findings underscore the photocatalyst’s stability and resilience under repeated operational cycles.

Nickel leaching was assessed after each stability cycle of the photocatalyst. Across all experimental tests, no detectable leaching was observed, confirming the successful incorporation of nickel into the titania matrix and demonstrating the sample’s stability under operational conditions. Supplementary Figures S2 and S3 provide images of the EDTA solution at the end of each stability cycle, along with the corresponding absorbance spectra. The results clearly indicate that nickel leaching was negligible in all experimental tests.

#### 3.2.3. Influence of Photocatalyst Dosage

**Figure 7:**
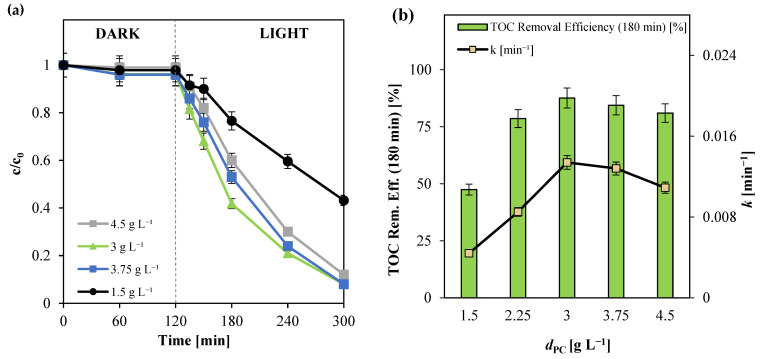
(**a**) Paracetamol degradation registered for different photocatalyst dosages; (**b**) TOC removal efficiency after 180 min of irradiation and apparent kinetic degradation constant values obtained for different photocatalyst dosages.

Given the superior photocatalytic performance of Ni(0.1%)-TiO_2_ in the degradation and mineralization of paracetamol, this sample was selected for subsequent experimental investigations. The effect of photocatalyst dosage on pollutant degradation was examined to determine the optimal operating conditions. Photocatalyst dosage plays a pivotal role in photocatalytic processes as it directly influences the availability of active sites and the photon absorption capacity, both of which are critical determinants of photodegradation efficiency [53,77]. As shown in Figure 7, the optimal photocatalyst dosage was found to be 3 g L^−1^. At this concentration, maximum degradation and mineralization efficiencies were achieved, with an apparent degradation kinetic constant of 0.0134 min^−1^. The improved photodegradation rates at this dosage can be attributed to the increased absorption of photons, which effectively enhanced the generation of reactive species [78,79].

However, when the photocatalyst dosage was increased beyond the optimal level to 4.5 g L^−1^, a decline in degradation and mineralization efficiencies was observed. This reduction can be explained by the increased opacity of the solution, which impeded photon penetration within the reactor, thereby limiting light absorption despite the abundance of active sites [80,81]. These findings highlight the importance of optimizing photocatalyst dosage to balance photon absorption and minimize light scattering for enhanced photocatalytic performance.

#### 3.2.4. Influence of Initial Paracetamol Concentration (c_0_)

**Figure 8:**
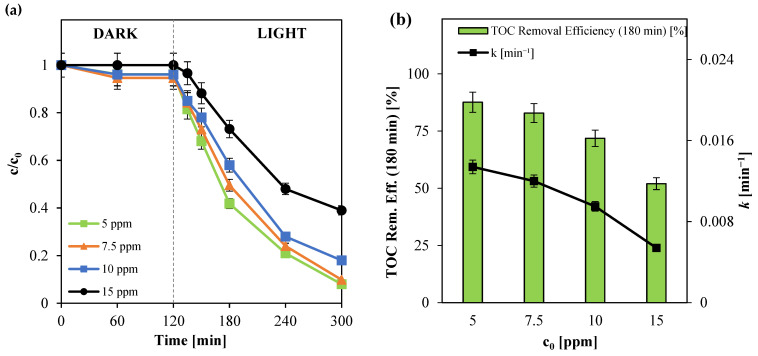
(**a**) Paracetamol degradation for different initial pollutant concentrations; (**b**) TOC removal efficiency after 180 min of irradiation and apparent kinetic degradation constant values obtained for different initial paracetamol concentrations.

The effect of initial paracetamol concentration on its photocatalytic degradation and mineralization was systematically investigated using Ni(0.1%)-TiO_2_ as the photocatalyst. Experiments were conducted with initial paracetamol concentrations ranging from 5 to 15 ppm, employing the optimal photocatalyst dosage of 3 g L^−1^, as determined in previous tests. The corresponding results are illustrated in Figure 8.

The maximum photocatalytic activity was observed at an initial paracetamol concentration of 5 ppm, achieving a degradation efficiency of approximately 92% (Figure 7a) and a total organic carbon (TOC) removal of 88% (Figure 7b) after 180 min of visible light irradiation. As anticipated, degradation efficiency was inversely proportional to the initial pollutant concentration, with higher concentrations leading to reduced photocatalytic performance. This decline in efficiency at elevated concentrations can be attributed to competitive adsorption dynamics on the catalyst surface [82,83]. The increased paracetamol adsorption restricted the availability of active sites for water and oxygen molecules, thereby limiting the formation of reactive hydroxyl and superoxide radicals essential for effective photocatalysis [83,84]. Consequently, mineralization efficiency decreased. Notably, at an initial concentration of 5 ppm, the degradation process followed pseudo-first-order kinetics, yielding an apparent kinetic constant of 0.0134 min^−1^.

#### 3.2.5. Investigation of Reactive Oxygen Species in the Photocatalytic Degradation of Paracetamol Using Ni(0.1%)-TiO_2_

The photocatalytic activity of the Ni(0.1%)-TiO_2_ photocatalyst was conducted to elucidate the role of reactive oxygen species (ROS) in the degradation of paracetamol under visible light irradiation, using the optimal operative conditions identified in previous tests (initial paracetamol concentration = 5 ppm, photocatalyst dosage = 3 g L^−1^). Specific scavenger molecules were employed to discern the contributions of individual ROS: isopropanol (IPA, 10 mM) for hydroxyl radicals (OH^•^) [53], benzoquinone (BQ, 1 μM) for superoxide anions (O_2_^•−^) [57], disodium ethylenediaminetetraacetate (EDTA, 10 mM) for positive holes (h^+^) [85], and potassium bromate (KBrO_3_, 1 mM) for other reactive oxygen species [86].

**Figure 9:**
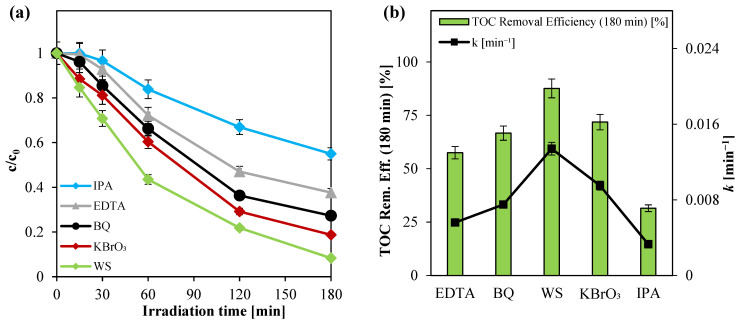
(**a**) Paracetamol degradation registered for the scavenger tests; (**b**) TOC removal efficiency after 180 min of irradiation and apparent kinetic degradation constant values obtained from the scavenger tests.

The results presented in Figure 9 reveal a significant reduction in photocatalytic activity upon the inhibition of hydroxyl radical formation, while more moderate decreases were observed when scavenging superoxide anions and positive holes. Notably, in the presence of IPA, the apparent degradation rate constant for paracetamol decreased by approximately 75% compared to control experiments without scavengers. In contrast, the use of EDTA and BQ reduced the paracetamol mineralization rate by approximately 60% and 55%, respectively. These findings underscore the pivotal role of hydroxyl radicals in the photocatalytic degradation process.

**Figure 10:**
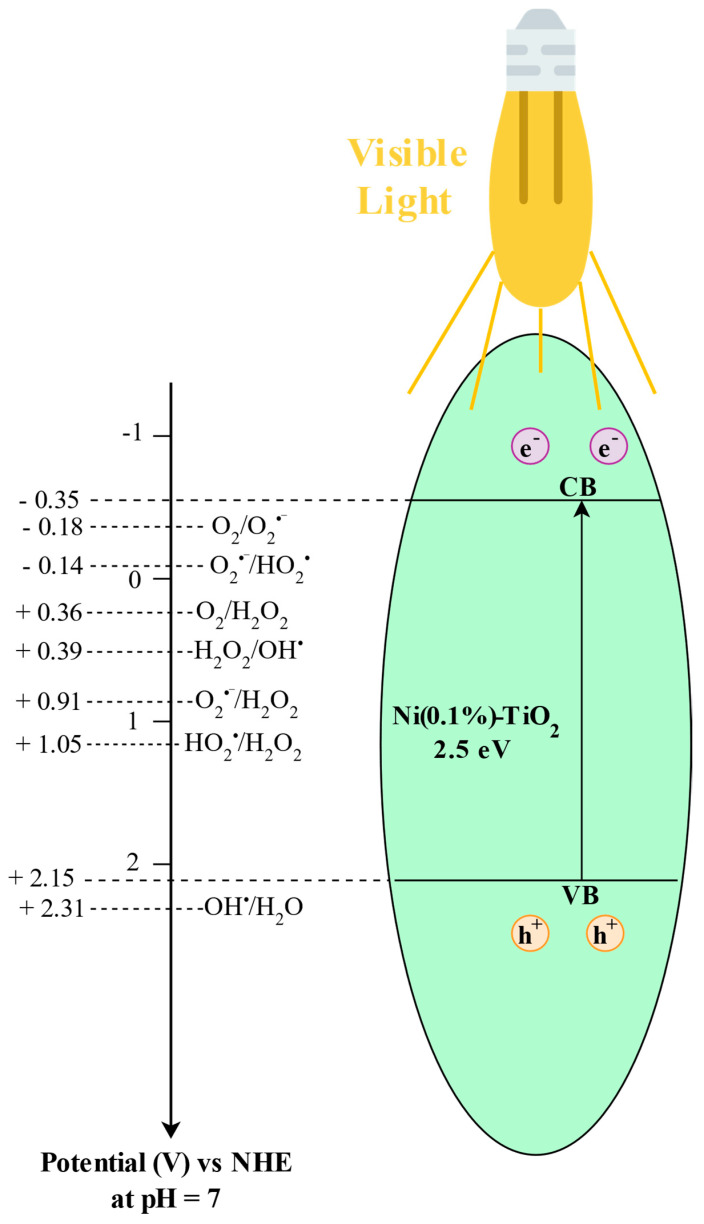
Proposed mechanism for charge carrier generation and reactive oxygen species (ROS) formation on the surface of Ni(0.1%)-TiO_2_ during the photocatalytic mineralization of paracetamol under visible light irradiation.

The photocatalytic mineralization mechanism of paracetamol over Ni(0.1%)-TiO_2_ can be elucidated by analyzing its electronic band structure and the thermodynamic feasibility of reactive oxygen species (ROS) formation. Based on Mulliken electronegativity theory, the estimated conduction band (*E*_CB_) and valence band (*E*_VB_) edge potentials of Ni(0.1%)-TiO_2_ are –0.35 V and +2.15 V versus the normal hydrogen electrode (NHE) at pH 7, respectively (Figure 10).

As evidenced by the UV–Vis diffuse reflectance spectroscopy (UV-Vis DRS) spectra (Figure 3), Ni(0.1%)-TiO_2_ exhibits visible light absorption, leading to the generation of photoinduced electron-hole pairs (Equation (10)). The photoexcited electrons in the conduction band possess sufficient reducing power to convert adsorbed molecular oxygen (O_2_) into superoxide radical anions (O_2_^•−^), as the redox potential of the O_2_/O_2_^•−^ couple (–0.18 V vs. NHE) [87] is more positive than the *E*_CB_ of Ni(0.1%)-TiO_2_ (–0.35 V) (Equation (11)). These superoxide radicals can participate in subsequent reduction steps to generate hydrogen peroxide (O_2_^•−^/H_2_O_2_, +0.91 V vs. NHE) [87] (Equation (12)). At the same time, conduction band electrons may directly reduce molecular oxygen to H_2_O_2_ via a two-electron pathway (O_2_/H_2_O_2_, +0.36 V vs. NHE) [87], facilitated by proton-coupled electron transfer mechanisms such as proton hopping and surface diffusion (Equation (13)). Furthermore, superoxide radicals can react with hydronium ions to form hydroperoxyl radicals (O_2_^•−^/HO_2_^•^, –0.14 V vs. NHE) [87], which can subsequently generate hydrogen peroxide (HO_2_^•^/H_2_O_2_, +1.05 V vs. NHE) [87,88] on the catalyst surface (Equations (14) and (15)). Hydrogen peroxide is further reduced by conduction band electrons to form highly reactive hydroxyl radicals (H_2_O_2_/OH^•^, +0.39 V vs. NHE) [87] (Equation (16)), which are primarily responsible for the oxidative degradation and mineralization of paracetamol, as confirmed by scavenger tests (Equation (17)).

Simultaneously, the photogenerated holes in the valence band are not sufficiently oxidative to generate hydroxyl radicals from water (OH^•^/H_2_O, +2.31 V vs. NHE) [87], given that the *E*_VB_ (+2.15 V) is less positive than the required redox potential. However, these holes can directly oxidize paracetamol molecules (Equation (17)), contributing significantly to its degradation and mineralization, as previously reported in the literature [89]. Therefore, the photocatalytic performance of Ni(0.1%)-TiO_2_ can be attributed to the synergistic generation of ROS through interfacial electron transfer processes. Electrons contribute to the formation of superoxide H_2_O_2_, while holes are involved in direct pollutant degradation. The observed results (Figure 8) strongly suggest that paracetamol mineralization is predominantly driven by hydroxyl radicals, which are generated both through direct oxidation by VB holes and via secondary reactions involving H_2_O_2_ formed at the catalyst surface.

Based on these observations, the proposed mechanism for the mineralization of paracetamol involves the sequential generation of hydroxyl radicals via electron and hole interactions [90,91], as detailed in the following pathway:

(10) $$Ni\left(0.1\%\right)-{TiO}_{2}+h\nu \to h^{+}+e^{-}$$

(11) $$O_{2}+e^{-}\to O_{2}^{-•}$$

(12) $$O_{2}^{•-}+e^{-}+2H^{+}\to H_{2}O_{2}$$

(13) $$O_{2}+2e^{-}+2H^{+}\to H_{2}O_{2}$$

(14) $$O_{2}^{•-}+H^{+}\to {HO}_{2}^{•}$$

(15) $${HO}_{2}^{•}+e^{-}+H^{+}\to H_{2}O_{2}$$

(16) $$H_{2}O_{2}+e^{-}+H^{+}\to OH^{•}+H_{2}O$$

(17) $$Paracetamol+OH^{•},h^{+}\to intermediates\to CO_{2}+H_{2}O$$

(18) $$e^{-}+h^{+}\to E+N$$

#### 3.2.6. Effect of Water Matrix Nature on Photocatalytic Activity

The presence of inorganic ions in water is known to significantly influence photocatalytic processes by altering reaction dynamics and reactive species availability [92,93]. To evaluate this effect, additional experiments were conducted using tap water (Table 3) spiked with paracetamol as a model contaminant. These investigations aimed to assess the impact of the water matrix on photocatalytic degradation efficiency under visible light irradiation.

**Figure 11:**
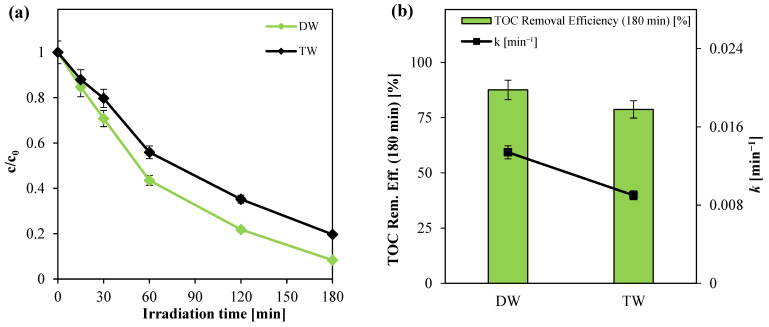
(**a**) Paracetamol degradation registered for the tests with different water matrix nature; (**b**) TOC removal efficiency after 180 min of irradiation and apparent kinetic degradation constant values obtained from tests with different water matrix nature.

The results, depicted in Figure 11, indicate that the photocatalytic system effectively degraded and mineralized paracetamol even in the presence of the complex tap water matrix. After 180 min of visible light exposure, the mineralization efficiency reached 78% in tap water (TW), compared to approximately 88% in distilled water (DW). However, the degradation kinetics in tap water exhibited a noticeable decline, with the apparent rate constant being approximately 33% lower than that observed in distilled water. This reduction can be attributed to the presence of inorganic ions in tap water, which likely act as radical scavengers, thereby decreasing the availability of photogenerated reactive oxygen species (ROS) essential for efficient photocatalytic degradation [52]. These findings underscore the importance of accounting for water matrix composition in the design and optimization of photocatalytic systems for practical environmental applications.

#### 3.2.7. Evaluation of Electrical Energy Consumption and Comparison of Photocatalytic Degradation of Paracetamol from Literature Studies

The electrical energy consumption associated with the photodegradation of 90% of paracetamol in 1 m^3^ of water contaminated with the pharmaceutical compound was assessed under optimal operating conditions. This analysis was conducted using the following equations derived from the work of Bolton et al. (Equation (19)) [94]:

(19) $$E_{E/O}=\frac{P\;t_{90\%}\;1000\;\frac{L}{m^{3}}}{V\;\ln\left(\frac{c\left(t_{0}\right)}{c\left(t\right)}\right)\;60\;\frac{min}{h}}$$

(20) $$f_{c}=\frac{1000\frac{L}{m^{3}}}{60\;\frac{min}{h}}$$

(21) $$k=\frac{\ln\left(\frac{c\left(t_{0}\right)}{c\left(t\right)}\right)}{t_{90\%}}$$

(22) $$k_{sp}=\frac{k}{P}$$

(23) $$E_{E/O}=\frac{f_{c}}{V\;k_{sp}}$$

where *P* represents the nominal power of the light source (kW), *t*_90%_ is the irradiation time required to achieve a 90% reduction of paracetamol (min), *V* is the treated solution volume (L), *c*(*t*_0_) is the initial concentration of paracetamol (ppm), *c*(*t*) is the paracetamol concentration at the irradiation time *t* (ppm), $f_{c}$ is the dimensional correction factor, and *k_sp_* is the specific apparent kinetic constant for paracetamol degradation with respect to the electrical power supplied to the light source for the activation of the photocatalyst.

Subsequently, several studies from the literature have been reviewed regarding the photocatalytic degradation of paracetamol in aqueous solutions:

- W1: the photocatalytic activity of ZnO/GQDs/CdSe composite under visible light was investigated for paracetamol degradation [95]. A 50 mg portion of the synthesized photocatalyst was dispersed in 200 mL of a pollutant solution with a final concentration of 10 mg L^−1^. The mixture was stirred in complete darkness for 60 min to establish adsorption–desorption equilibrium. Subsequently, the system was exposed to visible light under an intensity of 75 W.
- W2: the photocatalytic performance of the ZnO/gC_3_N_4_ photocatalyst was assessed for the degradation of paracetamol under visible light irradiation in a photo-reactor equipped with a Xenon light source of 500 W power fitted with 420 nm UV cutoff filter [96]. The reaction involved a 100 mL paracetamol solution at an initial concentration of approximately 30 ppm.
- W3: The Ba_0.95_Bi_0.05_Fe_0.95_Cu_0.05_O_3_ photocatalyst was tested for paracetamol degradation under visible light (metal halide efficacy lamp, 244 W, HQI- T250/Daylight, OSRAM GmbH, Germany) [97]. The photocatalyst concentration was 0.75 g L^−1^, and the initial contaminant concentration was 20 ppm.
- W4: the use of ZnO/AgNPs composite in the degradation of paracetamol was examined in a slurry reactor under simulated solar light with an irradiance of ~1000 W m^−2^ (Sciencetech SS1.6 kW, Canada) [98]. The system treated a 3.5 mL solution of paracetamol with an initial contaminant concentration of 5 ppm.
- W5: the effects of 40% Pr/Bi_4_V_2_O_11_ on paracetamol degradation were studied using a 200 mL solution at an initial pollutant concentration of 10 ppm, irradiated by a visible light source (300 W) [99].
- W6: the photocatalytic efficiency of the chitosan-supported covalent organic framework (CSCF) photocatalyst was assessed for the degradation of paracetamol under visible light irradiation [100]. A 60 mL solution containing 3 ppm of paracetamol was treated with CSCF in a double-walled reactor maintained at room temperature. Prior to illumination, the solution was stirred in darkness for 30 min to ensure adsorption–desorption equilibrium. Visible light from a 300 W Xenon lamp equipped with a UV cutoff filter (λ ≥ 420 nm) was then applied, achieving a paracetamol degradation efficiency of 99.8% within 180 min.

From the data provided in these six studies, the degradation kinetic constants for paracetamol were determined. Additionally, the electrical energy consumption required to achieve 90% pollutant reduction in 1 m^3^ of solution was calculated using Equation (23) and compared to the energy requirements of the photocatalytic system investigated in this study under optimal conditions. The results are presented in Table 4. A comparison with literature studies reveals that the nickel-doped TiO_2_, activated by visible light, significantly reduces energy consumption for treating paracetamol-contaminated aqueous solutions, achieving a consumption of 148.1 kWh m^−3^.

## 4. Conclusions

The photocatalytic performance of Ni-doped TiO_2_ photocatalysts was evaluated for the degradation and mineralization of paracetamol in aqueous solutions under visible light irradiation. All samples were synthesized using the sol-gel method, and their physicochemical properties were thoroughly characterized using Raman spectroscopy, UV–Vis diffuse reflectance spectroscopy (UV–Vis DRS), and surface area measurements. UV–Vis DRS analysis revealed that doping TiO_2_ with Ni significantly enhanced its optical absorption in the visible light region, narrowing the band gap compared to undoped TiO_2_ (3.11 eV). Specifically, the band gap decreased from 2.53 eV to 2.49 eV as the nickel concentration increased from 0.05% to 0.20%. Raman spectroscopy confirmed that anatase was the dominant crystalline phase in all prepared photocatalysts, with Ni successfully integrated into the TiO_2_ lattice. Additionally, the specific surface area of the doped catalysts was higher than that of pure TiO_2_ (101 m^2^ g^−1^), with the Ni(0.1%)-TiO_2_ composite exhibiting a surface area of 103 m^2^ g^−1^. The photocatalytic activity of the Ni-TiO_2_ composites was assessed for paracetamol degradation under visible light (>400 nm). The Ni(0.1%)-TiO_2_ photocatalyst demonstrated the highest performance, achieving 88% mineralization of paracetamol after 180 min of visible light exposure. To assess the catalyst’s stability, five consecutive tests were conducted, showing that its performance remained stable over time. After the same 180 min irradiation period, the mineralization efficiency was sustained at approximately 84%, with an apparent kinetic rate constant of 0.010 min^−1^ for paracetamol degradation. Furthermore, the influence of catalyst dosage and initial paracetamol concentration on the photocatalytic process was investigated. The optimal catalyst dosage was determined to be 3 g L^−1^, while the optimal initial paracetamol concentration was found to be 5 ppm. Hydroxyl radicals, generated under visible light, were identified as the primary reactive oxygen species responsible for the degradation of paracetamol.

The Ni(0.1%)-TiO_2_ photocatalyst also exhibited effective performance in paracetamol removal from tap water, with a total organic carbon (TOC) removal efficiency of 78.8% after 180 min. Additionally, the photocatalytic system demonstrated significantly lower energy consumption compared to similar photocatalytic systems reported in the literature for paracetamol degradation. In conclusion, the Ni(0.1%)-TiO_2_ photocatalyst exhibits significant potential for the efficient, cost-effective removal of pharmaceutical pollutants under visible light. Its straightforward synthesis method not only enhances its photocatalytic performance but also ensures its feasibility for large-scale industrial applications.

## Figures and Tables

**Table 1:** Amount of nickel(II) acetate tetrahydrate and TTIP used for the preparation of Ni-doped TiO_2_ photocatalysts.

Photocatalyst	*m*_Ni(OCOCH_3_)2·4H2O_ [g]	*m*_TTIP_ [g]	*n*_Ni_/*n*_TiO2_
TiO_2_	0	12	
Ni(0.05%)-TiO_2_	0.00725	12	0.00069
Ni(0.10%)-TiO_2_	0.0145	12	0.00138
Ni(0.15%)-TiO_2_	0.02175	12	0.00207
Ni(0.20%)-TiO_2_	0.029	12	0.00276

**Table 2:** Results of specific surface area (SSA), energy bandgap (*E*_bg_), and point of zero charge (PZC).

Photocatalyst	SSA [m^2^ g^−1^]	*E*_bg_ [eV]	PZC
TiO_2_	101	3.11	5.95
Ni(0.05%)-TiO_2_	102	2.53	5.75
Ni(0.10%)-TiO_2_	103	2.5	5.47
Ni(0.15%)-TiO_2_	103.5	2.44	5.18
Ni(0.20%)-TiO_2_	105	2.49	4.95

**Table 3:** List of the physicochemical properties of tap water.

Parameter	Value
Conductivity (µS cm^−1^)	370
Sodium (ppm)	3.16
Potassium (ppm)	1.08
Calcium (ppm)	59.8
Magnesium (ppm)	12.9
Chlorides, Cl^−^ (ppm)	6
Sulfates, SO_4_^2−^ (ppm)	3.4
Bicarbonates, HCO_3_^−^ (ppm)	249
Nitrates, NO_3_^−^ (ppm)	7.1

**Table 4:** Comparison of the electrical energy costs for degradation of 90% of paracetamol in 1 m^3^ of solution for our system with Ni(0.1%)-TiO_2_ versus other studies reported in the literature.

Photocatalyst	Type of Light	*P* [kW]	*k_sp_* [min^−1^ kW^−1^]	*V* [L]	*E_E/O_* [kWh m^−3^]	Reference
ZnO/GQDs/CdSe	Visible Light	0.075	0.533	0.2	156.3	[95]
ZnO/gC_3_N_4_	Visible Light	0.5	0.100	0.1	1666.7	[96]
Ba_0.95_Bi_0.05_Fe_0.95_Cu_0.05_O_3_	Visible Light	0.244	0.135	0.1	1232.3	[97]
ZnO/AgNPs	Simulated Solar Light	1.6	0.005	0.0035	9.52 × 10^5^	[98]
40% Pr/Bi_4_V_2_O_11_	Visible Light	0.3	0.025	0.2	3289.5	[99]
CSCF	Visible Light	0.3	0.113	0.06	2451.0	[100]
Ni(0.1%)-TiO_2_	Visible Light	0.012	1.125	0.1	148.1	Our reaction system

## Data Availability

Data will be provided on request to the corresponding author.

## Supplementary Materials

The following supporting information can be downloaded at: https://www.mdpi.com/article/10.3390/nano15070530/s1, Figure S1: Band gap calculation by UV–VIS DRS spectra for: (a) TiO_2_, (b) Ni(0.05%)-TiO_2_, (c) Ni(0.10%)-TiO_2_, (d) Ni(0.15%)-TiO_2_, and (e) Ni(0.20%)-TiO_2_; Figure S2: Images of the solution after the addition of EDTA for the evaluation of nickel leaching at the end of the (a) first, (b) second, (c) third, (d) fourth, and (e) fifth stability cycle; Figure S3: Absorbance spectra (200–900 nm) of the solution after the addition of EDTA for the evaluation of nickel leaching at the end of five stability cycles.
