# Supplementary material for: Synthesis and Characterization of a Novel Sol–Gel-Derived Ni-Doped TiO2 Photocatalyst for Rapid Visible Light-Driven Mineralization of Paracetamol
Source: Nanomaterials (Basel). 2025 Mar 31;15(7):530. doi: 10.3390/nano15070530 (PMC11990163; doi:10.3390/nano15070530)
Supplement: Supplementary file 1 [file nanomaterials-15-00530-s001.zip › nanomaterials-3522782-supplementary.pdf]

nanomaterials

Supplementary Materials

Synthesis and Characterization of a Novel Sol–Gel-Derived Ni-Doped TiO_2 Photocatalyst for Rapid Visible-Light-Driven Mineralization of Paracetamol

Nicola Morante, Katia Monzillo, Vincenzo Vaiano and Diana Sannino *

Supplementary Materials

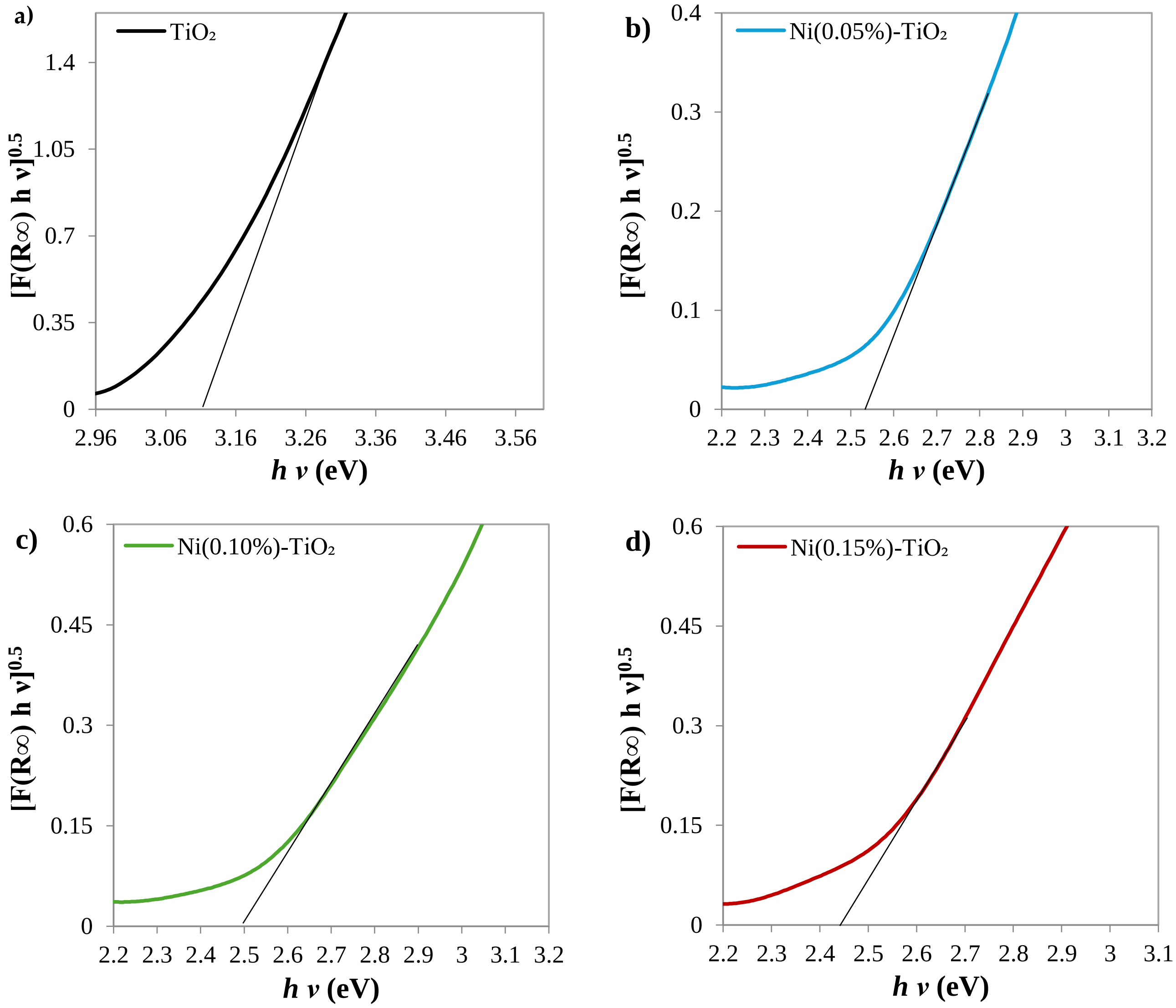

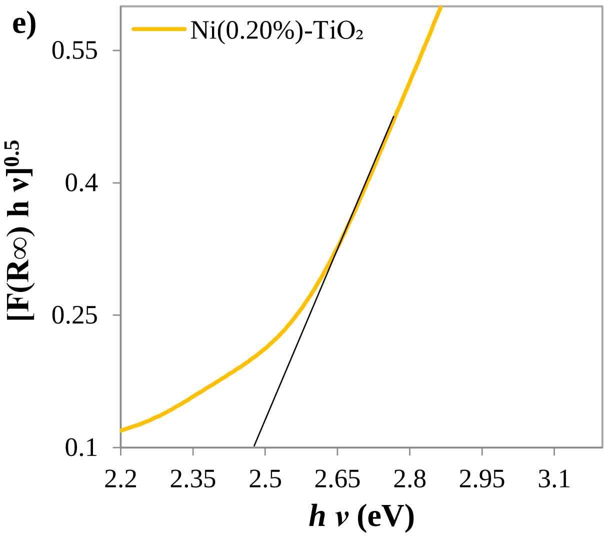

Figure S1: band gap calculation by UV–VIS DRS spectra for: a) TiO_2, b) Ni(0.05%)-TiO_2, c) Ni(0.10%)-TiO_2, d) Ni(0.15%)-TiO_2 and e) Ni(0.20%)-TiO_2.

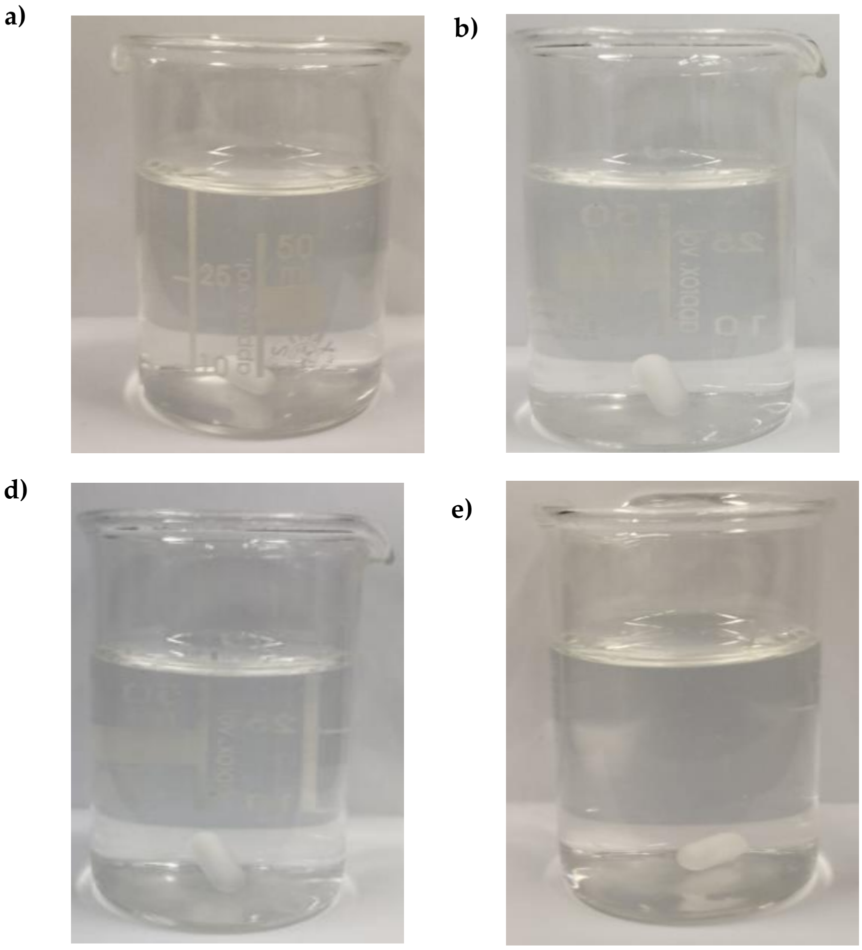

Figure S2: Images of the solution after the addition of EDTA for the evaluation of nickel leaching at the end of the a) first, b) second, c) third, d) fourth, and e) fifth stability cycle.

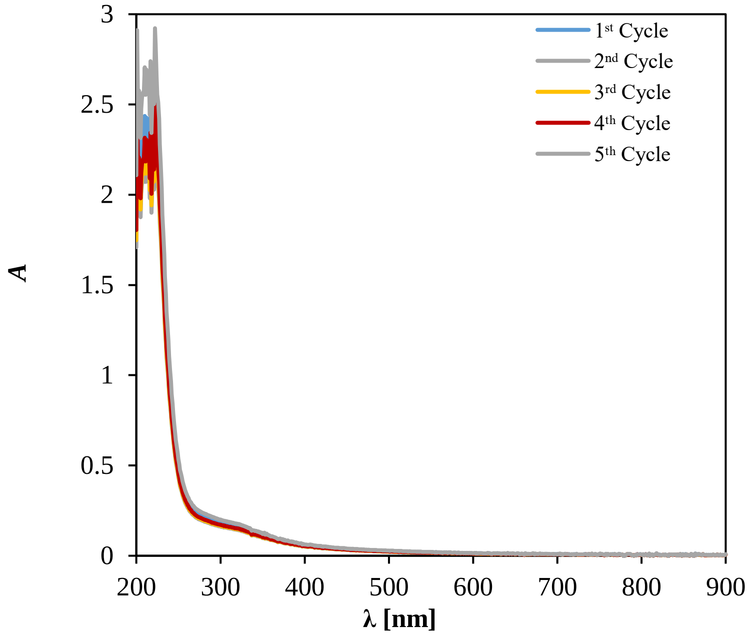

Figure S3: Absorbance spectra in the range 200-900 nm of the solution after the addition of EDTA for the evaluation of nickel leaching at the end of the five stability cycles.
